# Lemierre’s disease: a case with bilateral iliopsoas abscesses and a literature review

**DOI:** 10.1186/1749-7922-9-38

**Published:** 2014-05-15

**Authors:** Nicholas TE Bird, Derek Cocker, Paul Cullis, Richard Schofield, Ben Challoner, Alastair Hayes, Martin Brett

**Affiliations:** 1 General Surgery, University Hospital Aintree, Lower Lane, Liverpool L97AL, UK; 2 Infectious Diseases, Royal Liverpool Hospital, Prescot Street, Liverpool L7 8XP, UK; 3 General Surgery, Royal Liverpool Hospital, Prescot Street, Liverpool L7 8XP, UK; 4 General Surgery, Warrington Hospital, Lovely Lane, Warrington WA5 1QG, UK; 5 General Surgery, Royal Infirmary of Edinburgh, 51 Little France Crescent Old Dalkeith Road, Edinburgh EH16 4SA, UK

**Keywords:** Lemierre, Fusobacterium, Fusobacterium Necrophorum, Bilateral, Iliopsoas abscess

## Abstract

Lemierre’s disease is characterized by sepsis, often with an oropharyngeal source, secondary septic emboli and internal jugular vein thrombosis (Lancet 1:701–3, 1936. Clin Microbiol Rev 20(4):622–59, 2007). Septic emboli affecting many bodily sites have been reported, including the lungs, joints, bones, and brain. The case report describes an unusual case of Lemierre’s disease in a 64 year old gentleman causing profound sepsis, acute kidney injury, bilateral iliopsoas abscesses and a right hand abscess. To our knowledge, this is the first reported case of Lemierre’s disease in the context of bilateral psoas abscesses, and highlights the ambiguity surrounding the definition of Lemierre’s disease. The clinical literature review highlights the difficulty in definitively diagnosing the condition and offers some suggestions for recognising and refining the diagnostic criterion of Lemierre’s.

## Background

In 1936, Lemierre described a series of lethal anaerobic septicaemias caused by the anaerobic bacterium known today as *Fusobacterium necrophorum.* A group of these patients suffered from “postanginal septicaemias”, characterized by internal jugular vein thrombosis and septic emboli with a focus within the head and neck [1]*.* Septic emboli affecting many body sites have been reported, including the lungs, joints, bones, liver, brain and meninges. Septic metastasis to muscle has been described but is relatively rare [2-5]. This case report describes an unusual case of Lemierre’s disease in a 64 year old gentleman causing profound sepsis, acute kidney injury, bilateral iliopsoas abscesses and a right hand abscess [6-76].

### Case presentation

A 64 year old gentleman presented to the A&E department of a district general hospital with lethargy, fever and lumbar back pain radiating to the groins of two days duration. His background included hypertension managed with multiple medications, diet controlled type 2 diabetes mellitus, chronic back pain, latent peptic ulcer disease, and bilateral total hip and knee arthroplasties. On examination, he was hypoxic (94% oxygen saturation), hypothermic (35.6°C) and tachycardic with new onset, fast atrial fibrillation (rate 142/minute), but normotensive. In addition, he was diffusely tender in the supra-pubic region and in both loins, especially on the right. Neurological examination was normal other than MRC grade 4/5 power in the lower limbs.

Blood tests demonstrated a marked inflammatory response with raised CRP (373 mg/L) and predominantly neutrophilic leucocytosis (20.5 × 10^9^/L). Acute kidney injury (urea 31.4 mmol/L; creatinine 244 μmol/L) and mildy deranged liver function tests (alkaline phosphatase 343 IU/L; GGT 183 IU/L; ALT 52 IU/L; bilirubin 14 μmol/L) were evident. Arterial blood gases demonstrated a metabolic acidosis (pH 7.32; base excess −8 mEq/L). A chest radiograph was normal. Urinalysis was positive for leucocytes and erythrocytes only. Blood cultures were taken and broad spectrum antibiotics were commenced for presumed urosepsis.

**Figure 1:**
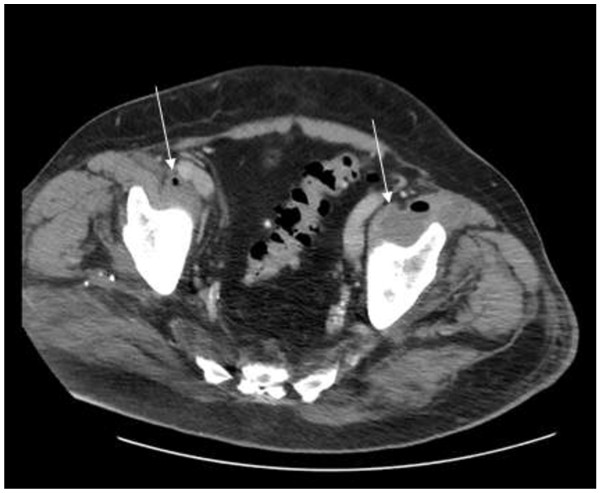
Transverse view on CT of the bilateral iliopsoas abscesses.

**Figure 2:**
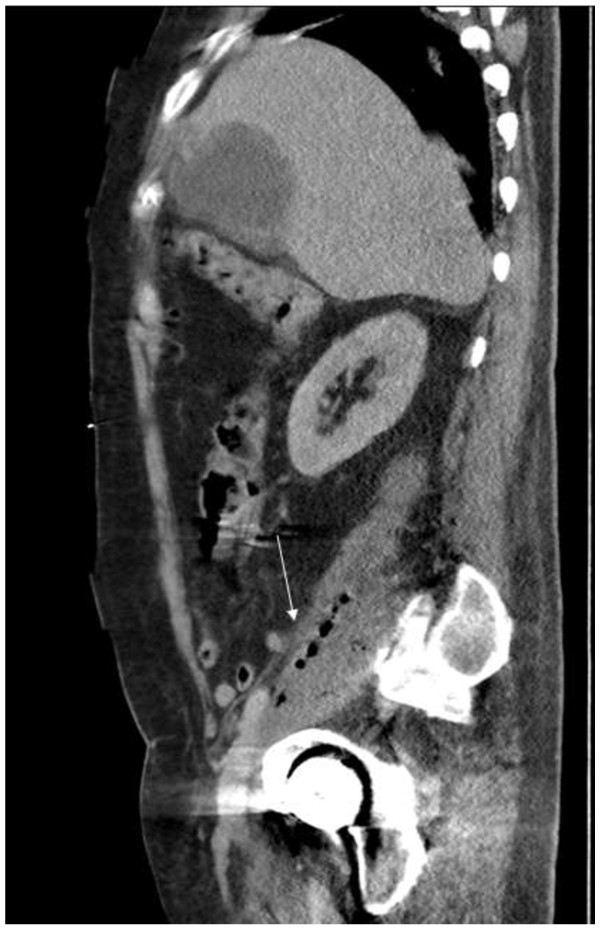
CT demonstrated Sagittal View of Abdomen and Pelvis demonstrating gas locules in Right Iliopsoas Region.

24 hours after admission, the right hand became diffusely swollen, erythematous and tender, and the patient continued to experience pyrexia. His urine cultures yielded *Serratia marcescens* sensitive to the antibiotics. Ultrasonography of the urinary tract failed to demonstrate hydronephrosis. Ultrasonography of the right hand showed generalised soft tissue oedema with a 1 cm deep fluid filled collection containing echogenic material overlying the MCP joints.The following day, the acute kidney injury worsened (urea 43.4 mmol/L; creatinine 351 μmol/L). An urgent CT thorax/abdomen/pelvis demonstrated an unexpected finding of bilateral iliopsoas abscesses, most extensive on the right side which contained a considerable volume of gas (Figures 1 and 2).

The patient proceeded to theatre for drainage of the abscesses. During intubation the anaesthetist noted the oropharynx was sloughy and inflamed and accordingly biopsies were taken. Bilateral groin incisions were used to approach the iliopsoas muscles in the extra-peritoneal plane. On the right side the abscess cavity involved the entire length of the iliopsoas muscle and contained 100 ml of cream coloured pus as well as gas. On the left side an estimated 40 ml of pus was contained within the lower psoas muscle. There was no evidence of communication with the replaced hip joints on either side. Drains were placed into the cavities. The hand abscess was also drained and samples from all sites were sent to microbiology. The patient was then transferred post-operatively to ICU for inotropic support (noradrenaline) and ongoing fluid resuscitation.

**Figure 3:**
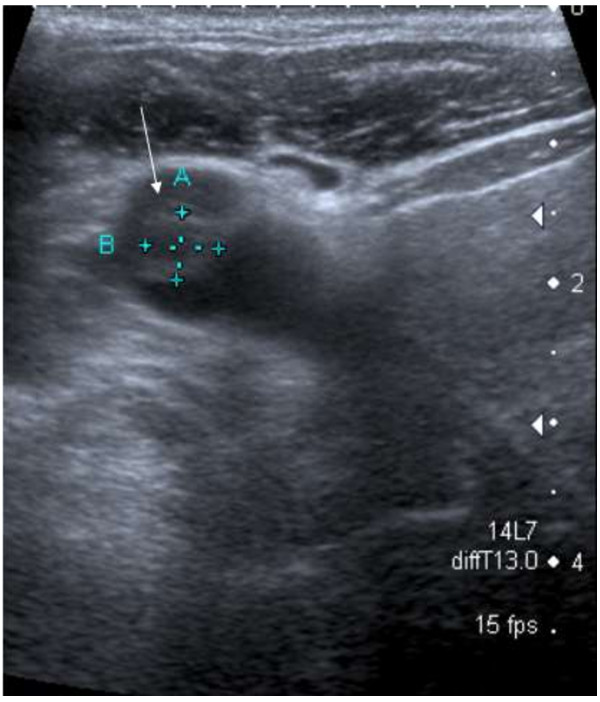
>50% occlusive right internal jugular vein thrombus on ultrasonography.

72 hours after admission the blood cultures returned a yield of *F. necrophorum* and subsequently tazocin and metronidazole were commenced. He remained intubated for a further three days. An echocardiogram was largely unremarkable. The oropharyngeal biopsies demonstrated, particularly in the vallecula, acute-on-chronic infection but no discrete microbial growth was achieved. The other microbiological samples did not yield any growth on extended culture runs. Subsequent neck ultrasonography confirmed a partially occlusive right internal jugular vein thrombus at the subclavian confluence (Figure 3). A CT neck/thorax confirmed this but did not demonstrate other occult pathology. Anticoagulation therapy with warfarin was subsequently commenced. The patient is now well and not suffering from any residual disability.

## Discussion

Despite reports of human illnesses caused by what is now known as *F. necrophorum* appearing within early 20^th^ Century literature, the consensus definition of Lemierre’s syndrome remains unclear [5,77]. The authors undertook a literature review to further clarify these diagnostic criteria. Using the PubMed search engine we utilised the following mesh headings: Lemierre’s (All Text); and Fusobacterium (All Text); and Case (Title/Abstract).

The search yielded 96 papers published since 1980 from a wide global geographical area inclusive of Asia, South America, North America and Europe. The authors used only papers which had symptomatic descriptions, bacteriological evidence, radiological evidence and descriptions in English which could possibly demonstrate a definitive diagnosis of Lemierre’s disease. This left 78 identifiable cases in the literature.

Analysis of the 78 cases demonstrates that the oropharynx tends to be the primary infective site with 59/78 (77% - see Table 1) of all cases demonstrating symptoms prior to sepsis of an acute oropharyngeal infection. 16/78 (21%) of the remaining cases had primary infective sites from other anatomical locations. 5/78 (6%) of these cases originated in the ears with symptoms of otitis externa occurring prior to widespread sepsis. 3/78 (4%) cases originated in the soft tissues in the neck from originally superficial infections of the skin in both the anterior (2/3 cases) and the posterior (1/3 cases) triangles. 3/78 (4%) of cases had syndromic components but no obvious primary infective site.

**Table 1:** Site of primary infection

	***Oropharynx***	***Cranio-facial***	***Extra cranio-facial***	***Unknown***
***Number of cases reported***	*N = 59*	*N = 13*	*N = 3*	*N = 3*
		5 Ear	1 Spine	
		5 Dental	1 Uterus	
		3 Neck	1 Hand	

A particularly contentious aspect is whether or not the presence of thrombophlebitis of the internal jugular vein is essential in the diagnosis [77]. In our case, ultrasound and CT confirmed the presence of substantial internal jugular vein (IJV) thrombus. Our literature review demonstrated 54/78 (69% - see Table 2) of reported cases had thrombus in the IJV. In 2/78 (3%) of cases the IJV thrombus propagated cranially resulting in thrombophlebitis of the cranial veins. In both cases there were headache symptoms but no dynamic neurology. 18/78 (23%) of patients had thrombophlebitis in other anatomical locations which correlated with an ipsilateral local primary infective site. An unusual example of this is a case of thrombophlebitis of the ovarian vein in a case of fusobacterial sepsis from an Intra-Uterine Device [30]. 4/18 (22%) of these cases involved peritonsillar abscesses with ipsilateral facial vein involvement and substantial cellulitis of the face and neck region. 6/18 (33%) of cases of alternative anatomical sites for thrombophlebitis were the great veins of the cranium with the cavernous sinus and the sigmoid sinus being involved individually in 1/18 (5%) cases respectively. There were 2/18 (11%) cases of clot propagation distally from the cavernous sinus to the sigmoid sinuses. 1/18 (5%) cases demonstrated thrombophlebitis in the vasculature near the site of infective metastasis indicating that fusobacterial sepsis produces a highly pro-coagulant inflammatory response in patients [60]. This effect is demonstrated in the 2/78 (3%) cases where there was thrombus formation within the carotid artery [45,61], a vessel with typically very high laminar flows which, according to Virchow’s triad, would preclude against clot formation and aggregation.

**Table 2:** Site of thrombus formation

	***Internal jugular vein***	***Alternative vessel***	***Negative for thrombus***	***Unknown***
***Number of cases reported***	*N = 54*	*N = 22*	*N = 3*	*N = 8*
		6 Sigmoid Sinus		
		4 Facial Vein		
		4 Cavernous Sinus		
		2 External Jugular Vein		
		2 Carotid Artery		
		1 Subclavian Vein		
		1 Axillary Vein		
		1 Hepatic Vein		
		1 Ovarian Vein		

The 78 individual patients produced 105 metastatic abscess sites indicating that multiple sites of metastases are a common feature of Lemierre’s syndrome (see Table 3). The most common site of metastasis is to the lungs which occurred in 55/105 (52%) metastatic sites and 55/78 (70%) of individual cases. 10/105 (9%) of metastases occurred in other soft tissue areas, of which 7/10 (70%) had concomitant pulmonary metastases. 6/105 (5%) metastatic sites were in the joints with 2/6 (33%) of these having associated pulmonary metastases. 18/105 (17%) metastases were to solid organs and bones. 12/18 (69%) soft tissue metastases occurred with pulmonary metastases. Therefore 21/55 (37%) of patients with pulmonary metastases developed further metastatic abscesses throughout the body with the most common metastases being to solid organs in 12/21 cases (60%). 12/18 (67%) of the metastases were to the cranial vault including cerebral (4/12), subdural (3/12) and epidural (2/12) anatomical locations. 4/12 (33%) of the cranial vault metastases had no pulmonary metastases. All of these cases had extensive cranial vein thrombophlebitis and, in 1 case, carotid artery thrombus.

**Table 3:** Site of metastatic spread and progression to surgery

	***Pulmonary***	***Soft tissue***	***Articular***	***Solid organ/bone***
***Number of cases reported***	*N = 55*	*N = 10*	*N = 6*	*N = 22*
		2 Mediastinal	2 Hip	10 CNS
		1 Masseter	1 TMJ	3Endocardial
		1 Parotid	1 C1/C2	3 Liver
		1 Shoulder	1 Knee	2 Orbital
		1Ear	1 Shoulder	2 Solid Bone
		1 Neck		1 Kidney
		1 Subphrenic		1 Pericardial
		1 Leg		
***Progression to surgery***	9	2	1	4
***Mortality***	2	0	0	2

The primary method of treatment of Lemierre’s Syndrome is through the use of broad-spectrum antimicrobials and supportive treatment. Surgery was utilised as a treatment modality in 24/78 (31%) cases in an attempt to gain source control in patients with refractory sepsis. Despite the presence of extensive pulmonary metastases which would make anaesthesia more dangerous, the surgical cohort had a 0% mortality rate while the overall cohort had a mortality rate of 4/78 (5%). 3 of the fatal cases were at the extremes of age, being 79 [18], 80 [50] and 10 years old respectively [43], with multiple metastatic sites and severe sepsis. The remaining fatality was a 34 year old gentleman with a delayed presentation to hospital one week post-onset of systemic symptoms with metronidazole resistant fusobacterial sepsis and multiple metastatic sites including heart valve vegetations [14]. Although this cohort is small it would seem to indicate that the outcomes are poorer for patients with reduced physiological reserve, locally advanced inflammation and multiple metastatic sites.

## Conclusion

Riordan has previously highlighted the epistemological difficulty in definitively diagnosing Lemierre’s as a distinct disease entity [77]. Indeed there are numerous terms and diagnostic classifications utilised inchoately by multiple authors but Riordan argues that Lemierre’s should be confined to *fusobacterium necrophorum* sepsis originating in the oropharynx. While we cannot conclusively prove that in our case profound fusobacterial sepsis originated as a consequence of oropharyngeal infection, the biopsies taken of the oropharynx do demonstrate an acute-on-chronic inflammation which would fit with the subsequent clinical manifestation of Lemierre’s Syndrome. The anaerobic blood cultures grew *fusobacterium necrophorum* which is the vital component for a diagnosis of Lemierre’s disease and is the only consistent component of the three general terms of necrobacillosis, post-anginal sepsis and Lemierre’s syndrome utilised in the medical literature.

The presence of substantial IJV thrombosis in our case, while consistent with the literature, is controversial with respect to the fact that the patient had had a central venous catheter inserted for 3 days on ICU prior to appropriate radiological investigations of the neck and therefore the provenance of the thrombus is contestable. There is debatable evidence regarding the length of time a central venous catheter needs to be in situ before occlusive thrombus forms. Some studies have suggested that less than 3 days with a central catheter in-situ can cause small thrombus formation [6,7]. However other equally robust studies have suggested that a central venous catheter needs to be in-situ for approximately 11 days to develop extensive occlusive thrombus [8] as demonstrated in our case. The literature review demonstrated that 31% of all cases did not have thrombosis of the IJV, however there were only 3/78 (4%) of cases with no associated thrombosis. Therefore thrombosis in the presence of fusobacterial bacteraemia would be a more appropriate diagnostic criterion than defining the disease by specific anatomically located thromboses.

In the context of the literature our case was unusual in that it demonstrated unique anatomical variation of the metastases and required surgery as the primary modality of treatment. Our patient did not have any pulmonary metastases which some authors have argued is a key diagnostic criterion for Lemierre’s syndrome [5]. However, our literature review has demonstrated that 30% of the cases had no pulmonary involvement. In view of this fact the authors support Riordan’s suggestion that Lemierre’s Syndrome should be reconstituted as *fusobacterium necrophorum* sepsis, however with the additional diagnostic criterion of the presence of thrombosis. It would seem that the septic metastases are a common complication of the syndrome with huge anatomical variation and as such are not essential to diagnose the condition.

### Consent

Written informed consent was obtained from the patient for publication of this Case report and any accompanying images. A copy of the written consent is available for review by the Editor-in-Chief of this journal.

## Abbreviations

CRP: C - Reactive Protein; Mg/l: milligrams per litre; μmol/L: micromols per litre; mmol/L: millimols per litre; IU/L: International Units per litre; GGT: gamma glutamyl-transpeptidase; ALT: Alanine Aminotransferase; CT: Computed Tomography; MCP: Metacarpophalangeal; ICU: Intensive Care Unit; IJV: Internal Jugular Vein.

## Competing interests

The author declares that they have no competing interest.

## Authors’ contributions

NTEB: Recognised the uniqueness of presentation. Acquired background sources. Primary information analyst. Main writer of case. Read and approved the content of the case. PC: Additional background sources. Secondary writer. Read and approved the case content. DC: Additional background knowledge. Secondary writer. Proof read case. Edited for submission. BC: Additional background research and paper sourcing for literature review. RS: Image acquisition. Anonymised radiographic data. AH: Additional key source acquisition. Proof read and helped edit paper. MB: Consultant surgeon responsible for overall patient care and patient data. Read and approved manuscript. All authors read and approved the final manuscript.
